# A Siamese Swin-Unet for image change detection

**DOI:** 10.1038/s41598-024-54096-8

**Published:** 2024-02-25

**Authors:** Yizhuo Tang, Zhengtao Cao, Ningbo Guo, Mingyong Jiang

**Affiliations:** Space Engineering University, Beijing, China

**Keywords:** Change detection, Remote sensing, Swin transformer, Swin-Unet, Siamesenet, Electrical and electronic engineering, Computer science, Computational science, Environmental impact

## Abstract

The problem of change detection in remote sensing image processing is both difficult and important. It is extensively used in a variety of sectors, including land resource planning, monitoring and forecasting of agricultural plant health, and monitoring and assessment of natural disasters. Remote sensing images provide a large amount of long-term and fully covered data for earth environmental monitoring. A lot of progress has been made thanks to deep learning's quick development. But the majority of deep learning-based change detection techniques currently in use rely on the well-known Convolutional neural network (CNN). However, considering the locality of convolutional operation, CNN unable to master the interplay between global and distant semantic information. Some researches has employ Vision Transformer as a backbone in remote sensing field. Inspired by these researches, in this paper, we propose a network named Siam-Swin-Unet, which is a Siamesed pure Transformer with U-shape construction for remote sensing image change detection. Swin Transformer is a hierarchical vision transformer with shifted windows that can extract global feature. To learn local and global semantic feature information, the dual-time image are fed into Siam-Swin-Unet which is composed of Swin Transformer, Unet Siamesenet and two feature fusion module. Considered the Unet and Siamesenet are effective for change detection, We applied it to the model. The feature fusion module is designed for fusion of dual-time image features, and is efficient and low-compute confirmed by our experiments. Our network achieved 94.67 F1 on the CDD dataset (season varying).

## Introduction

Change detection (CD) aims to detect change area between dual-time remote sensing (RS) images. The change defined in RS image CD generally refers to the change of land cover or land use status, and detection is to achieve the purpose of identifying changes in specific areas through visual interpretation or related algorithms. CD plays a very important role in various practical applications, for instance, disaster evaluation, ecological environment detection, urban development planning and civil map revision.

The traditional RS image CD has the problems of complex procedures, low accuracy. Besides, the traditional methods require high-quality dual-time images. In this paper, we use ai algorithms to solve this problem. Because CD can be viewed as a unique task for semantic segmentation, we use the idea of semantic segmentation to do CD. The early semantic segmentation model was implemented by removing the full connection layer from the full convolutional neural network^1^ and adding deconvolution to restore the original resolution, but a lot of semantic information would be lost in this process. In deep learning, the deep convolution neural network based on U-Net^2^ is the most classic semantic segmentation network structure. U-Net^2^ is composed of skip connection structure and symmetric encoder and decoder. Through a succession of convolutions and down-samples operations, the encoder extract the features of the input image. The decoder recovers the resolution of the image through up-sampling and convolution, and the skip connection structure integrates the features of each layer in the process of down-sampling which alleviates the loss of spatial information. This technical path led several algorithms, including 3D U-Net^3^,Res-UNet^4^, U-Net +  + ^5^ and UNET3 + ^6^.Those algorithms developed for various semantic segmentation and CD tasks, and they are effective. So we apply U-net^2^ as part of our module.

The locality of convolution operations makes it challenging for models based on convolution operations to learn global semantic information, even though CNN-based models have produced good results, which makes such methods unable to completely meet the accuracy requirements of semantic segmentation and CD. Swin Transformer^7^ applies the Transformer^8^ structure that performs well in natural language processing(NLP) to the field of computer vision. Swin-Unet^9^ based on Swin Transformer block is a network look like U-shaped based solely on Swin Transformer^7^, with encoder, decoder, bottleneck, and skip connection. Like U-Net^2^ The Swin Unet structure is an ideal backbone for segmentation of RS images with only a few spatial information, and the self-attention structure's global feature extraction capabilities can also extract significant features from RS images.

Siamesenet^10^ is designed to measure how similar two inputs are.To conduct end-to-end detection, researchers develop a variety of fully convolutional networks in^11–13^ Recurrent neural networks and CNN are used in^14,15^ to extract characteristics from multi-temporal pictures. For CD with multisource VHR pictures, convolutional multiple-layer recurrent neural networks are also suggested^16^. These networks either use a two-stream structure to learn picture characteristics or combine two images into a single multi-channel input to do so. But in our model, the two pictures are passed through the network in turn with the same weights in the lowest layers, using a siamese architecture. The technique of learning common characteristics through shared and wholly same weights is fair because the two photographs were captured at separate times while still being in the same location. For CD, a siamese convolutional network is suggested in^17^. The model in^17^ utilizes a straightforward Euclidean distance-based thresholding segmentation independent from the model while combining the information collected by the siamese CNN. For better information fusion in our approach, deeper modules are designed.

Combining Swin Transformer^7^,U-Net^2^ and Siamesnet^10^, we designed a special network for RS image CD: Siam-Swin-Unet. Siam-Swin-Unet is made up of Siamesenet^10^, encoder, decoder, skip connection structure and feature fusion module. The encoder and decoder are both built based on the Swin Transformer block. The dual-time RS images are respectively processed by the Swin Transformer encoder with the same weights to extract features of dual-time images. The two feature maps are fused through the feature fusion module. The fused features are up-sampled by the Swin Transformer decoder with shared weights, and multi-scale features from the encoder are fused through the skip connection structure to perform CD task. Finally, the RS dual-time image features with resolution restored by the siamesenet are multiplied to ensure that the network uses the information extracted by the two siamesenet equally. Specifically, We can sum up our contributions as follows:

- Combining Siamesenet^10^ with the improved Swin-Unet, and applying it to RS image CD.
- The fusion features are obtained by adding the dual-time image features instead of using convolution, which reduce the model parameter.
- Dual-time image feature are up-sampled separately after feature fusion module1 with the two siamesenet to protect feature fusion.
- The impact of different Swin Transformer window sizes on CDD^18^ dataset CD performance is studied.

## Methods

In this section, we will introduce the Siam-Swin-Unet model, which is integrated by Siamesenet^10^, Swin Transformer^7^, U-Net^2^ and feature fusion modules. First, we'll describe the overall design of Siam-Swin-Unet, and then we will introduce Swin Transformer block, Siamesenet^10^, Patch Merging, Patch Expanding, feature fusion modules, and skip connection.

### Overall

**Figure 1:**
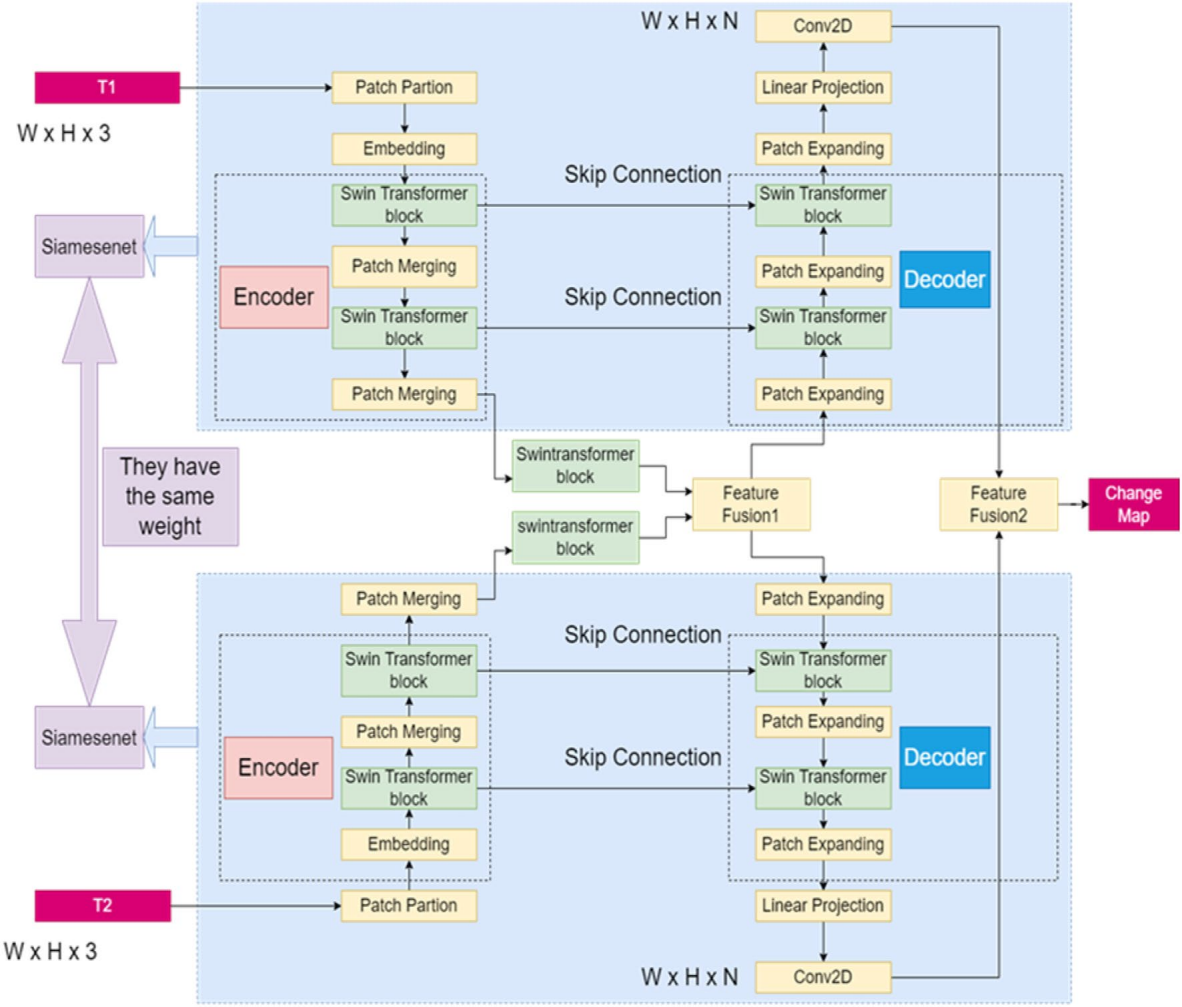
The architecture of the proposed Siam-Swin-Unet.

An overview of the model of Siame-Swin-Unet is displayed in Fig. 1. Siame-Swin-Unet is made up of siamesenet^10^, encoder, decoder, skip connection, feature fusion module 1 and feature fusion module 2. Among them, the basic building blocks of both encoder and decoder are Swin Transformer blocks, and the weight values are shared between the siamesenet. Dual-time images (W × H × 3) are inputted in the two siamese nets. The patch partition segments the input image into patches without overlapping to a size of 4 × 4. The number of channels through which the dual-time RS images were operated became 4 × 4 × 3 = 48. Moreover, the linear embedding layer maps the image features to a fixed number of channels (C). The purpose of Swin Transformer block is to learn the con-textual semantic information of input image and improve the model's global modeling ability for the image. The size does not change when the image goes through a Swin Transformer block. Patch merging down-samples images while increasing the quantity of feature channels of the model. Feature Fusion Module 1 uses summation to fuse dual-time image features. The encoder and decoder are symmetrical to each other, except that Patch Expanding, as opposed to Patch Merging, up-samples the feature to improve the resolution of the image. The skip connection is designed to utilize more spatial and details information from down-sampling. Furthermore, The skip connection operation can solve the problem of vanishing gradient. The last linear projection corresponds to the patch partition, which restores the image size to (W × H) by sampling the feature map up four times. Finally, the number of channels of the output image can be changed by a convolution operation so that the output of the model is (W × H × N). Thus, the two- or multi-classification tasks for RS image CD can be implemented. Finally, feature fusion module 2 uses multiplication to fuse features from two Siamesenets, which improves the model's effectiveness. Multiplication operations can combine multiple features or factors, allowing the model to make better use of multidimensional data. In many cases, there are certain associations between multiple features or factors in the data, and these associations can be taken into account through multiplication operations, so as to improve the prediction accuracy of the model.

In Fig. 1, T1 is the input image of the first moment, T2 is the input image of the second moment, the change map represents the area that changes or does not change between the T1 and T2 images. The white represents the changing area, and the black represents the unchanged area. Encoder is used to extract the features of the image, and the decoder is used to restore the resolution of the image. Patch Partion and Embedding are used to divide an image into small pieces and map them to a fixed dimension, which is equivalent to a convolution operation. Siamesenet, Swin Transformer block, Patch Merging, skip connection,conv2D and patch expanding will be introduced later.

### Siamesenet

**Figure 2:**
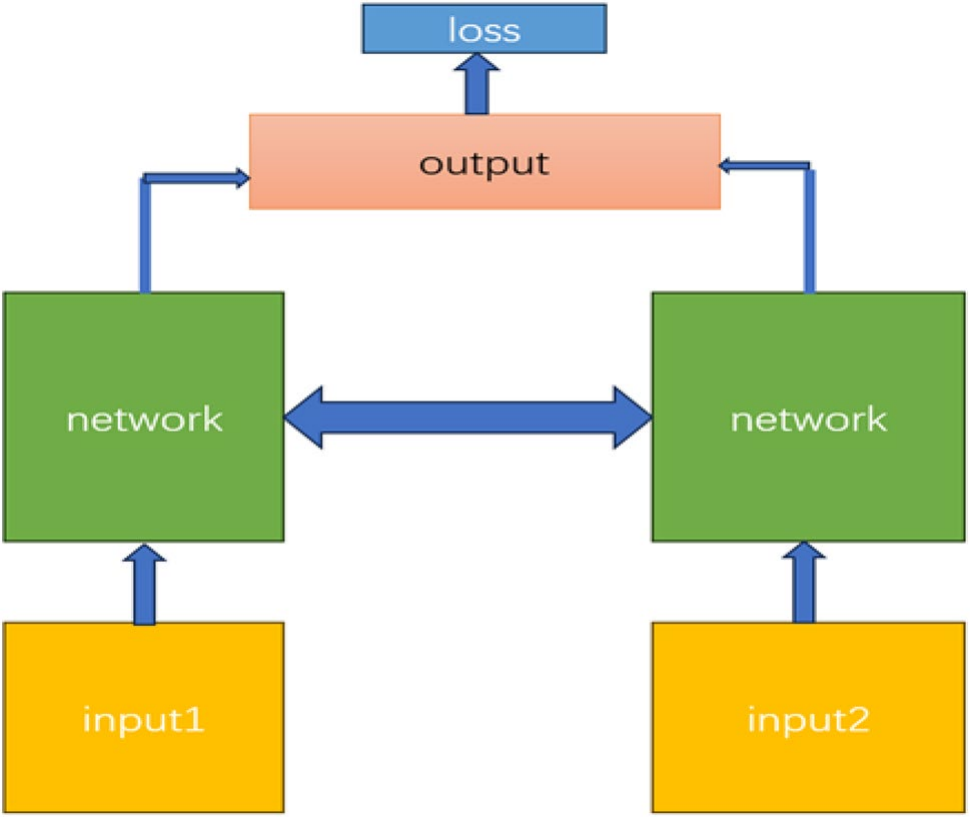
The idea of Siamesenet.

Siamesenet^10^ has a siamesed feature extraction network with shared and wholly same weights, which can extract the features of dual-time images separately and help the neural network generate Change Map. Currently, siamesenet^10^ has been used for CD of dual-time RS images. In this model, weights are shared among Siamesenets, which can extract features from dual-time RS images. In Siamesenet^10^ we conduct down-sample, up-sample, and finally the size of the feature map was restored to the same size of input. The idea of Siamesenet^10^ is illustrated in Fig. 2.

### Swin transformer block

**Figure 3:**
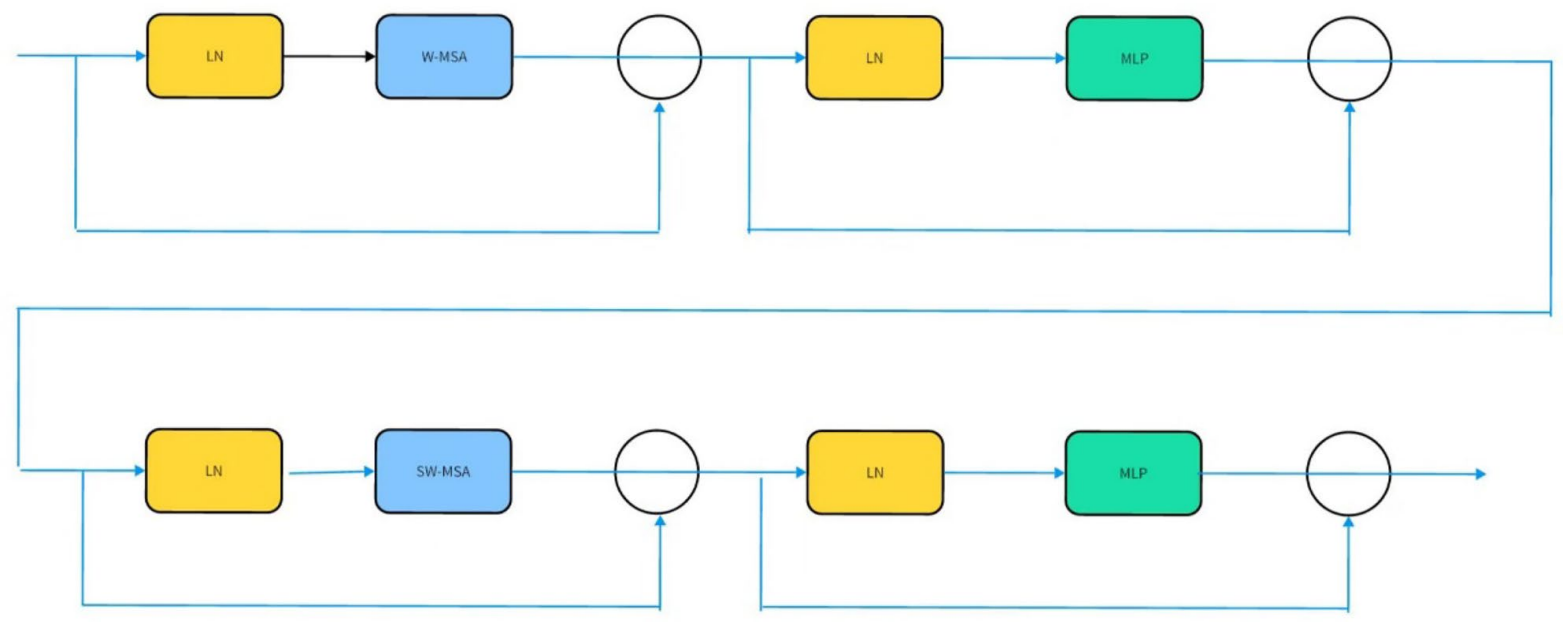
Swin transformer block.

The swin transformer block is consisted of several cells in Fig. 3.LN is a layer normalization operation. MLP is a Multi layer Perceptron. W-MSA and SW-MSA stand for window multi-head attention and shift window multi-head attention, respectively. To learn about context and semantics, The Swin transformer block compute the attention score in W-MSA and SW-MSA. To compute attention score in a small window can reduce computational complexity compared to the computing in the whole picture. And then, the shift window operation helps the network learn the attention information between the adjacent windows. The construction of the swin transformer block is displayed in Fig. 3.The calculation of attention can be explained in the following formula

1 $${\text{Attention}}({\text{Q,K,V}})={\text{SoftMax}}(\frac{{\text{Q}}\times {{\text{K}}}^{{\mathrm{T}}}}{\sqrt{{\text{d}}}}+{\mathrm{B}})\times {V}^{T}$$

**Figure 4:**
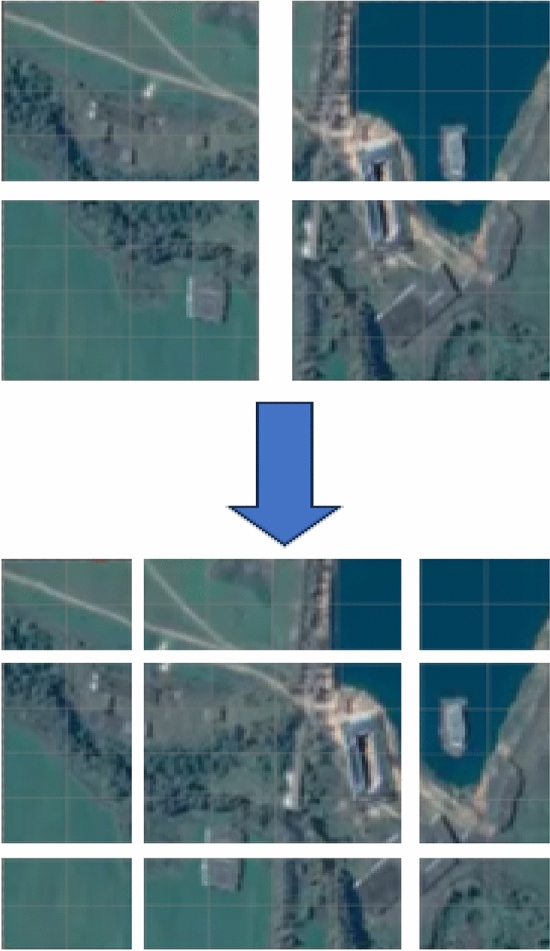
Mechanism of shift window.

The Q, K, V Respectively are query, key and value array. B are Relative position offset. The mechanism of the shift window is illustrate in Fig. 4 (Shift window is just a thought, it can be used as a square window or a free form rectangle. But our experiments were based on rectangular windows.).

### Patch merging

**Figure 5:**
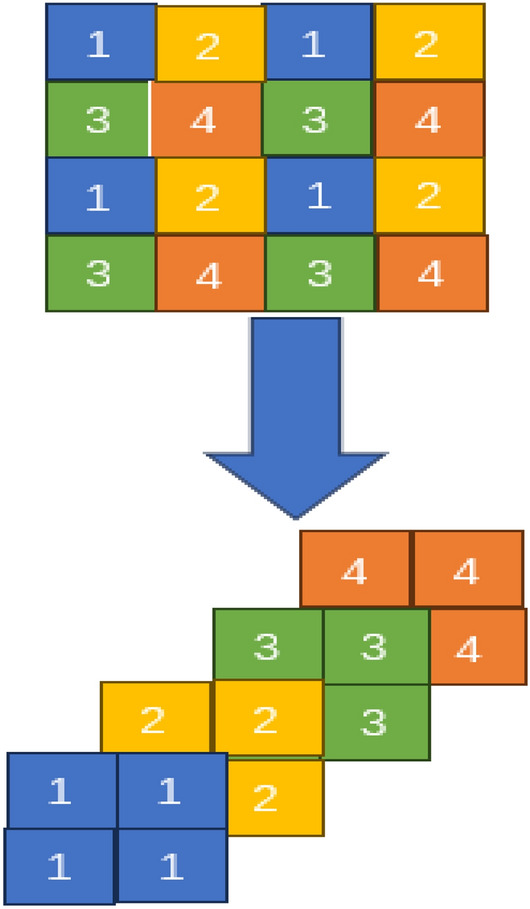
Mechanism of patch merging.

This module separates the input feature into 4 pieces, which are then concatenated. Such processing will cause a 2 × downsampling of the feature resolution. Additionally, as the concatenation procedure causes the feature dimension to increase to 4 × as much as the original, a linear layer is applied to the concatenated features in order to bring the feature dimension back to 2 × as much as the original.The idea of the patch merging is displayed in Fig. 5

### Skip connection

The multiresolution features come from the encoder are combined with the up-sampled data by the skip-connections, much like the U-Net^2^. To lessen the loss of spatial information due to downsampling, we combine the superficial features with the profound features. The dimension of the features after concatenation operation is kept constant with dimension of up-sampled features after a linear layer.

### Patch expanding

As an illustration, consider the first patch expanding layer. Prior to upsampling, The input features (W/16 × H/16 × C4) are given a linear layer to apply, which doubles the original feature dimension to (W/16 × H/16 × C8). Then, we apply the rearrange operation to lower the feature dimension to one-fourth of the input feature and extend the resolution of the input features to two times, which means from [(W/16 × H/16 × C8) to (W/8 × H/8 × C2)].

### Conv2D

The purpose of this module is to study a general network capable of performing tasks of two- and multi-classification change monitoring framework. Conv2D therefore converts the number of the channel of feature map to N. N represents the number of classifications.

### Ferture fusion module 1

The function of feature fusion module 1 is to fuse the feature maps of two dual-time RS images after down-sampling, and learn the semantic information that has changed in the two features. Conv3D can be used to fuse the two features, but it will make training more difficult. Consider that dual-time RS images are equally important. Therefore, this paper tries to fuse the dual-time RS image features with addition operation, which is equivalent to Conv3D with the same weight. It greatly reduces the computational complexity and training difficulty.

### Ferture fusion module 2

Feature Fusion Module 2 fuses two up-sampled feature maps, making full use of each layer of semantic information of the dual-time image, and further improve the network's ability to express changing features. Considering the need to improve the network's ability, we use multiplication for feature fusion. Multiplication can enhance the nonlinear representation of the model. Nonlinearity is an important feature in many datasets, and multiplication operations can introduce nonlinear factors to enable models to better handle nonlinear problems.

## Dataset

**Figure 6:**
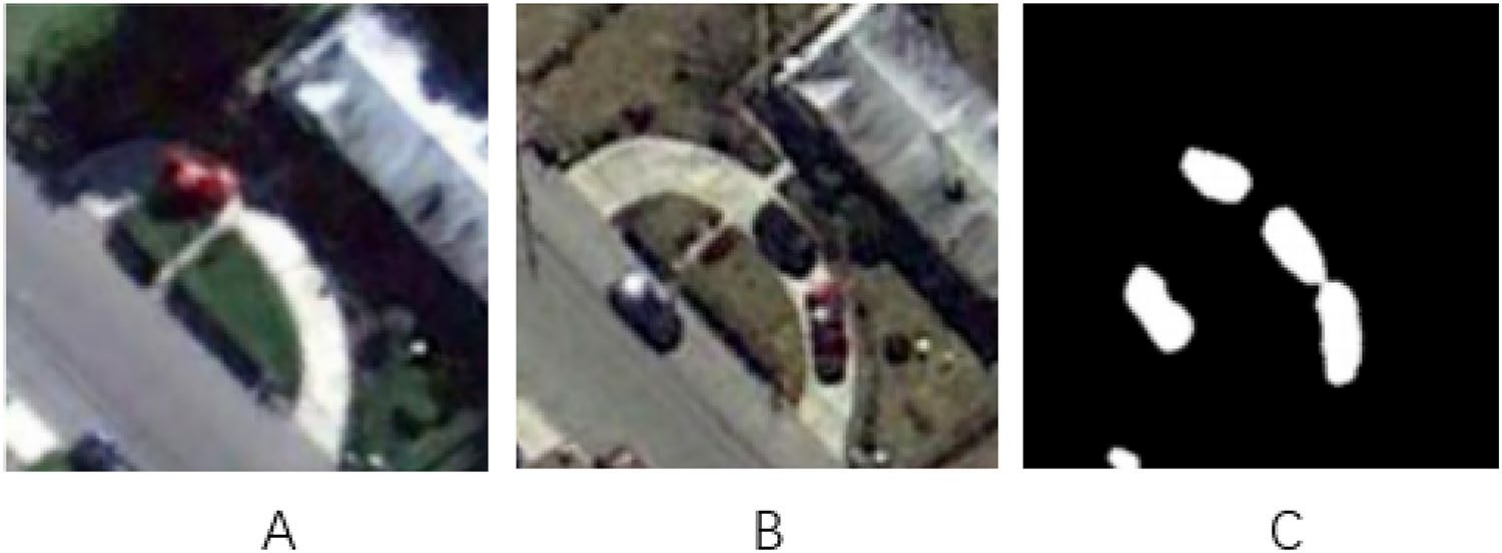
The example of the CDD dataset.

A dataset known as CDD^18^, one of the most used datasets in RS CD, was used to test this network. The CDD^18^ dataset comes from Google Earth images with a resolution of 3–100 cm and a size of 256 × 256 pixels. The dataset has 10,000 groups of training sets, 3000 groups of test sets and 3,000 groups of verification sets. There are various types of surface changes, including buildings, cars, land, roads and warehouses. The dataset has a long time span and contains data with large or small changes. The seasonal difference and illumination difference of RS images are considered, which can effectively detect the performance of Siam-Swin-Unet in detecting changes in RS images. The example of this dataset is illustrate in Fig. 6.The A is time1 image and B is time2 image, C is the label of change area. It is dichotomous class dataset, so the label is a binary image. The label help the network learn the weight.

## Experimental details

Siam-Swin-Unet is implemented based on python and pytorch. The GPU used in the test is NVIDIA TITAN X, and the memory is 24G. The super parameters of the training of the network are as follows: the number of model training epoch is 200, and the global optimal model is selected. The optimizer is Adam, and the initial learning rate is 1 × 10^(−4). Referring to the existing research, the loss function is a mixture of Jaccard Loss, Focal Loss,Dice Loss and Edge Loss. Among them, because the sample of CD task is extremely uneven, Diceloss can mitigate the adverse impact on backpropagation caused by the imbalance of CD sample, making the training unstable. Focal Loss can increase the weight of difficult to classify samples, making the model training more effective. Jaccard Loss is a kind of cross merger ratio loss, which can improve the IOU index of the model. Edge Loss can improve the edge details and further improve the accuracy of CD.

2 $$FocalLoss(p)=-a(1-p{)}^{\lambda }\mathit{log}(p)$$

3 $${\text{DiceLoss}}=\left.1-\frac{2|{\text{X}}\cap {\text{Y}}|}{|{\text{X}}|+|{\text{Y}}|}\right|$$

4 $$\mathrm{Jaccard Loss}=1-\frac{|{\text{X}}\cap {\text{Y}}|}{|{\text{X}}|+|{\text{Y}}|-|{\text{X}}\cap {\text{Y}}|}$$

5 $$\text{Edge Loss}=\frac{{\sum }_{{\text{x}}=1}^{{\text{W}}}\sum_{{\text{y}}=1}^{{\text{H}}}{{\text{E}}}_{{\text{i}},{\text{j}}}.(|{{\text{Y}}}_{{\text{i}},{\text{j}}}|-|{{\text{X}}}_{{\text{i}},{\text{j}}}|)}{{\text{W}}\times {\text{H}}}$$

In order to test the performance of the model, this paper uses confusion matrix as the evaluation method of the model performance. The evaluation index is F1. The higher the F1 value, the better the model performance. Formula of F1 and confusion matrix is as follows:

6 $$\begin{array}{ccc}confusion \,\, matreix& predict1& predict0\\ lable=1& TP& FN\\ lable=0& FP& TP\end{array}$$

7 $$F1=\frac{2TP}{2TP+FP+FN}$$

TP is True Positive, FP is False Positive and FN represent False Negative.

## Experiment results on CDD

**Figure 7:**
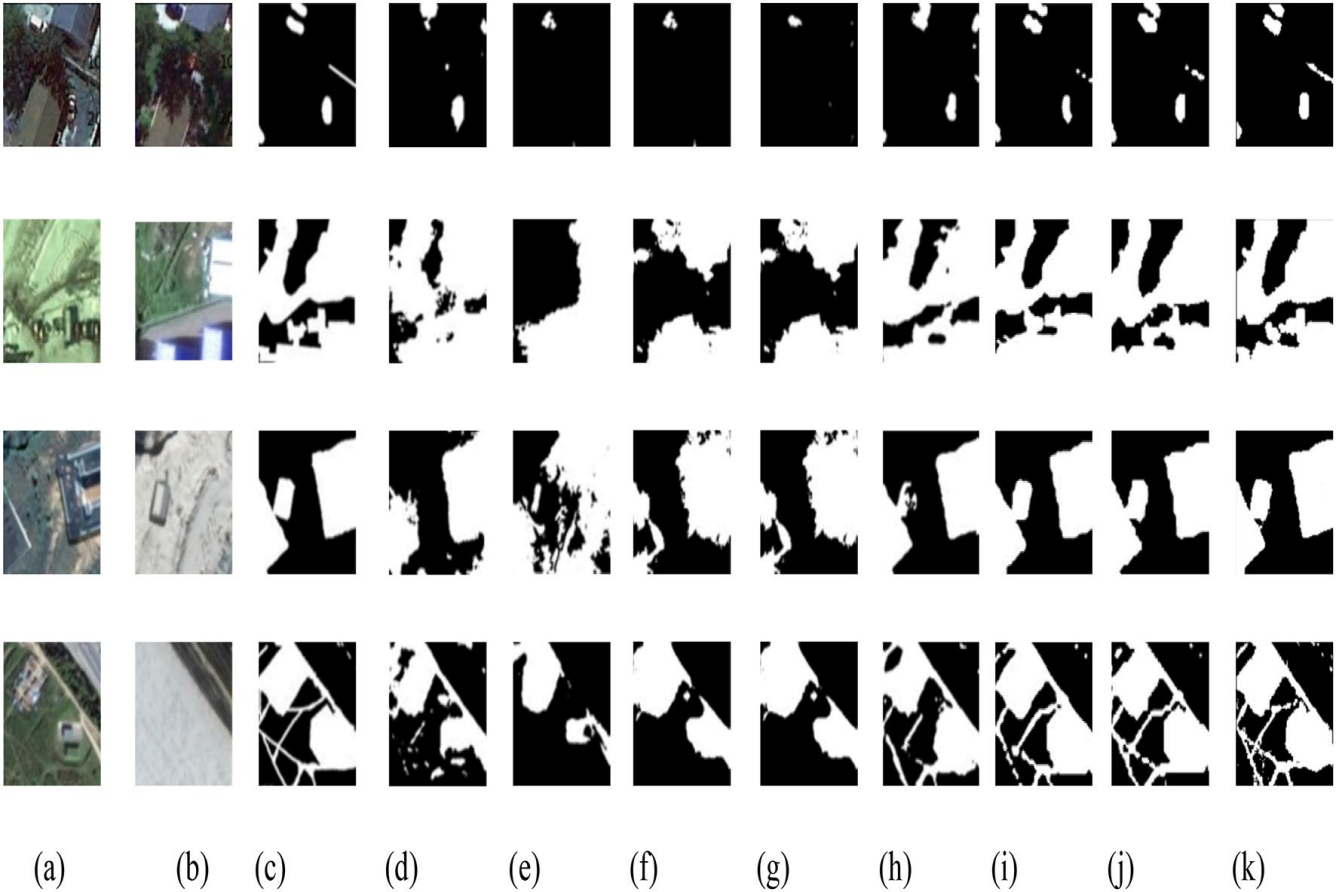
Results of several CD techniques on the CDD\* MERGEFORMAT^18^ dataset are visually compared: (**a**) The time1 image, (**b**) The time 2 image, (**c**) The label of the change area, (**d**) CDnet, (**e**) FC-EF,(**f**) FC-Siam-Diff, (**g**) FC-Siam- Con, (**h**) BiDateNet, (**i**) DASNet(VGG16), (**j**) DASN,(**k**) Siam-Swin-Unet.

A comparison of the performance of some models is shown in Table 1.The experimental results show that, in the CDD^18^ dataset, The F1 index of the model proposed in this paper is 94.67, which is superior to each comparison model. This proves the effectiveness of this model for CD. Through the Fig. 7, we can observed the output of Siam-Swin-Unet is closer to the label. To be more specific, The Siam-Swin-Unet is better at edge and detail detection and have Stronger capabilities to detect the change target.

**Table 1:** The F1 score of some networks.

Model	F1
CNnet	0.822
FC-EF	0.596
FC-Siam-Diff	0.691
FC-Siam-conc	0.687
BiDateNet	0.898
DSANet(VGG16)	0.919
DSANet(ResNet50)	0.927
Siame-Swin-Unet	0.9467

## Ablation study

We carried out ablation study to investigate the impact of various parameters on the model performance. Specifically, the impact of Window Size, Feature Fusion Module 1 on model performance will be disscussed below.

### Effect of window size

Swin Transformer blocks with different window sizes are applied to this model. We compare the effects of Window Size 4 and Window Size 8 on the model. In the Table 2, we can find that when the window is small, the CD performance is better, which is related to the size of the image in the dataset.

**Table 2:** The effect of windowsize.

Window size	Layers	Method	F1
4	3	Add	0.9467
8	3	Add	0.9281
4	3	Conv3D	0.9455
8	3	Conv3D	0.9258

### Effect of feature fusion module

Considering that the information contribution of dual-time RS images in CD should be the same, and the use of convolution layers will increase the training difficulty and computational complexity, for feature fusion module 1, we respectively use addition and Conv3D to fuse the features of dual-time RS images for comparison. In the Table 3, we can find that the simplest addition operation is directly used, and the effect of the model is better than that of a convolution operation.

**Table 3:** The effect of feature fusion module.

Method	Layers	Window size	F1
Conv3D	3	4	0.9467
Add	3	4	0.9281
Conv3D	3	8	0.9455
Add	3	8	0.9258

## Conclusion

In this article, we propose Siam-Swin-Unet that can perform dual-time RS image classification and multi classification CD tasks. The experimental results show that the use of additive operation to fuse the features of dual-time RS images instead of traditional convolution operations can retain the global semantic information of the features, reduce the model parameters without affecting the effect of the model. In addition, Though Siame-Swin-Unet does not perform any image enhancement operations on the dataset, the performance is very good, we get 94.67 points measured by F1 metrics. Next, we plan to use a feature fusion module that is more learnable and does not lose global semantic information for feature fusion. And try to add appropriate image enhancement module to further improve the prediction accuracy of the model.

## Data Availability

The datasets analysed during the current study are available in https://gitlab.citius.usc.es/hiperespectral/ChangeDetectionDataset.
